# PET imaging and pharmacological therapy targeting carbonic anhydrase-IX high-expressing tumors using US2 platform based on bivalent ureidosulfonamide

**DOI:** 10.1371/journal.pone.0243327

**Published:** 2020-12-09

**Authors:** Shimpei Iikuni, Hiroyuki Watanabe, Yoichi Shimizu, Yuji Nakamoto, Masahiro Ono

**Affiliations:** 1 Department of Patho-Functional Bioanalysis, Graduate School of Pharmaceutical Sciences, Kyoto University, Kyoto, Japan; 2 Department of Diagnostic Imaging and Nuclear Medicine, Graduate School of Medicine, Kyoto University, Kyoto, Japan; Biomedical Research Foundation, UNITED STATES

## Abstract

Carbonic anhydrase-IX (CA-IX) is attracting much attention as a target molecule for cancer treatment since high expression of CA-IX can lead to a poor prognosis of patients. We previously reported low-molecular-weight ^111^In/^90^Y complexes with a bivalent ureidosulfonamide scaffold ([^111^In/^90^Y]In/Y-US2) as cancer radiotheranostic agents for single photon emission computed tomography and radionuclide-based therapy targeting CA-IX. Here, we applied the US2 platform to positron emission tomography (PET) imaging and pharmacological therapy targeting CA-IX high-expressing tumors by introducing ^68^Ga and ^nat^In, respectively. In an *in vitro* cell binding assay, [^67^Ga]Ga-US2, an alternative complex of [^68^Ga]Ga-US2 with a longer half-life, markedly bound to CA-IX high-expressing (HT-29) cells compared with low-expressing (MDA-MB-231) cells. In a biodistribution study with HT-29 and MDA-MB-231 tumor-bearing mice, [^67^Ga]Ga-US2 showed accumulation in the HT-29 tumor (3.81% injected dose/g at 60 min postinjection) and clearance from the blood pool with time. PET with [^68^Ga]Ga-US2 clearly visualized the HT-29 tumor in model mice at 60 min postinjection. In addition, the administration of [^nat^In]In-US2 to HT-29 tumor-bearing mice led to tumor growth delay and prolonged mouse survival, while no critical toxicity was observed. These results indicate that [^68^Ga]Ga-US2 and [^nat^In]In-US2 may be useful imaging and therapeutic agents targeting CA-IX, respectively, and that US2 may serve as an effective cancer theranostic platform utilizing CA-IX.

## Introduction

Hypoxia in many kinds of solid tumors is caused by an imbalance between oxygen supply and consumption, and is closely associated with tumor propagation, malignant progression, and resistance to chemotherapy and radiotherapy [1–5]. Carbonic anhydrase (CA) isozymes, a class of zinc metalloenzymes, are found in most living organisms [5, 6]. CA-IX is the protein most markedly upregulated by hypoxia through the hypoxia inducible factor-1 (HIF-1) cascade, and is strongly associated with cancer progression [7–12]. CA-IX catalyzes the reversible hydration of carbon dioxide to a bicarbonate ion and a proton [7–12], which may promote cancer cell survival [13]. CA-IX is functionally involved in diverse aspects of cancer development, such as primary tumor growth, metastatic dissemination of cancer cells, and homing and growth of metastatic lesions [14]. Concerted regulation of the intra- and extra-cellular pH is conducted by CA-IX and various other proteins, such as aquaporins and anion exchangers [9–11, 15, 16]. The wild-type von Hippel Lindau (VHL) tumor suppressor protein binds to HIF-1 hydroxylated by oxygen, leading to the degradation of HIF-1 after ubiquitination. In other words, a deficit in the VHL gene, which is often observed in patients with clear cell renal cell carcinoma, contributes to HIF-1 stabilization, leading to high-level CA-IX expression [7–11]. CA-IX expression is limited in normal tissues except in the gastrointestinal tract [17]. In contrast, the expression of CA-IX is markedly increased in many types of tumors in response to hypoxia [5]. Moreover, CA-IX is pathologically expressed in cancer cells and located at the cell surface. Therefore, CA-IX has emerged as an attractive target for both the therapy and diagnosis of cancer.

Over the past few decades, many attempts to develop useful CA-IX imaging probes have been made. The chimeric monoclonal antibody cG250 (girentuximab), which is reactive with CA-IX, has been labeled with ^124^I, ^131^I, ^111^In, and ^89^Zr [18–22]. cG250 has shown potential for nuclear medical imaging; however, its slow clearance from the blood pool suggests limitations including a low signal-to-noise ratio on early imaging and a high toxicity due to the radiation burden. In contrast, small-molecule CA-IX imaging probes based on sulfonamide and coumarin have attracted much attention owing to their rapid pharmacokinetics and high affinity for CA-IX [23–33]. Moreover, an acetazolamide derivative, XYIMSR, labeled with ^111^In/^64^Cu for diagnostic imaging or ^177^Lu for therapy showed potentials for radiotheranostics targeting CA-IX-expressing tumors [29, 33, 34]. In addition to *in vivo* CA-IX imaging probes, many selective CA-IX small inhibitors based on scaffolds of sulfonamide [35, 36], ureidosulfonamide [30, 37–39], coumarin [40–42], and sulfocoumarin [43–45], have been reported. Among the large series of small-molecule inhibitors, SLC-0111 showed not only high affinity for CA-IX *in vitro*, but also promising anti-tumor properties in cancer models; moreover, clinical trials of SLC-0111 in subjects with advanced solid tumors have suggested its utility in cancer therapy [37–39].

**Fig 1:**
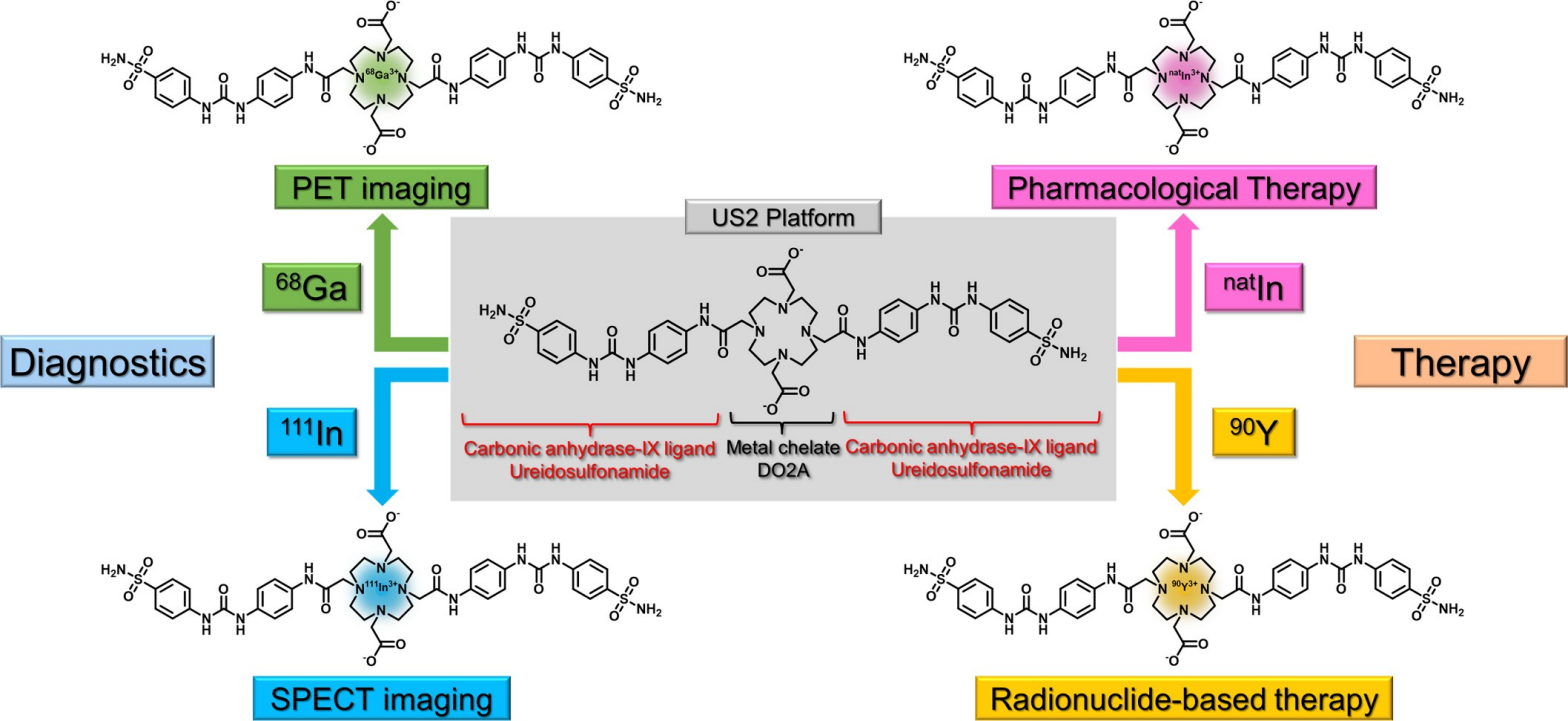
Concept of cancer theranostics with US2 platform utilizing CA-IX.

Recently, we reported an original theranostic platform based on ureidosulfonamide targeting CA-IX for cancer diagnosis and therapy (US2) (Fig 1) [46]. US2 consists of 1,4,7,10-tetraazacyclododecane-1,7-diacetic acid (DO2A) as the metal-chelating moiety and two ureidosulfonamide scaffolds, and is utilized to develop cancer diagnostic and therapeutic agents by conjugating with functional metals. US2 conjugated with ^111^In (γ-emitter) and ^90^Y (β^−^-emitter) ([^111^In]In-US2 and [^90^Y]Y-US2, respectively) were prepared for tumor single photon emission computed tomography (SPECT) imaging and radionuclide-based therapy targeting CA-IX, respectively. [^111^In]In-US2 showed high selectivity for CA-IX high-expressing (HT-29) cells *in vitro*, and demonstrated higher HT-29 tumor uptake at 1−4 h postinjection than other previously reported radioligands of CA-IX [26, 27, 47–52]. Moreover, *in vivo* SPECT imaging with [^111^In]In-US2 clearly visualized HT-29 tumors in model mice. In addition, [^90^Y]Y-US2 administration into HT-29 tumor-bearing mice significantly delayed tumor growth as compared with non-treated mice. Based on these encouraging results, in the present study, we utilized this US2 platform to develop novel positron emission tomography (PET) and pharmacologically therapeutic agents targeting CA-IX by conjugating with ^68^Ga (β^+^-emitter) and ^nat^In, respectively. ^68^Ga, the most widely utilized radiometal for PET, is available from a germanium-68/gallium-68 generator and easily conjugated with various metal chelators, such as DO2A. We synthesized [^68^Ga]Ga-US2 and evaluated its utility as a CA-IX imaging agent for PET. We performed fundamental evaluations with [^67^Ga]Ga-US2 due to the longer half-life of ^67^Ga (78 h) than that of ^68^Ga (68 min). In addition, tumor growth inhibition by the administration of ureidosulfonamide-based drugs, such as SLC-0111, has been reported by several groups [30, 37–39]. Therefore, we synthesized [^nat^In]In-US2, in which ^nat^In was introduced in order for it to exhibit a similar biodistribution to [^111^In]In-US2, and evaluated its utility for CA-IX high-expressing tumor therapy.

## Materials and methods

### General

All reagents were obtained commercially and used without further purification unless otherwise indicated. [^67^Ga]GaCl_3_ (0.3−0.6 N HCl solution) was purchased from FUJIFILM Toyama Chemical (Tokyo, Japan). [^68^Ga]GaCl_3_ was obtained from a commercial ^68^Ge/^68^Ga generator (IRE ELiT, Fleurus, Belgium) using 0.1 N HCl. ^1^H and ^13^C NMR spectra were recorded on JNM-ECS400 (JEOL, Tokyo, Japan) with tetramethylsilane as an internal standard. Coupling constants are reported in Hertz. Multiplicity was defined as singlet (s), doublet (d), or multiplet (m). High-resolution mass spectrometry (HRMS) was conducted with LCMS-IT-TOF (SHIMADZU, Kyoto, Japan). Reversed-phase high-performance liquid chromatography (RP-HPLC) was performed with a Shimadzu system (an LC-20AT pump with an SPD-20A UV detector, λ = 254 nm; SHIMADZU) with a Cosmosil C_18_ column (5C_18_-PAQ, 4.6 × 250 mm; Nacalai Tesque, Kyoto, Japan).

### Chemistry

The precursor for radiolabeling (US2) and corresponding ^nat^In complex ([^nat^In]In-US2) were synthesized according to our previous report [46].

#### Synthesis of [^nat^Ga]Ga-US2

To a solution of US2 (20 mg, 0.020 mmol) in dimethyl sulfoxide (DMSO) (1 mL) were added [^nat^Ga]Ga(NO_3_)_3_·nH_2_O (51 mg, 0.20 mmol) and 2-(*N*-morpholino)ethanesulfonic acid (MES) buffer (0.1 M, pH 5.5, 3 mL). The solution was stirred at 90°C for 6 h. After cooling to room temperature, the precipitate was filtered to give 8 mg of [^nat^Ga]Ga-US2 (38%). ^1^H NMR (400 MHz, DMSO-*d*_*6*_) *δ* 10.34 (s, 2H), 9.08 (s, 2H), 8.80 (s, 2H), 7.72 (d, *J* = 8.8 Hz, 4H), 7.60 (d, *J* = 8.8 Hz, 4H), 7.51 (d, *J* = 8.8 Hz, 4H), 7.42 (d, *J* = 8.8 Hz, 4H), 7.22 (s, 4H), 3.88−3.86 (m, 4H), 3.75−3.72 (m, 8H), 3.39−3.30 (m, 8H), 2.55−2.53 (m, 4H). ^13^C NMR (100 MHz, DMSO-*d*_*6*_) *δ* 170.0 (2C), 165.2 (2C), 152.3 (2C), 142.9 (2C), 136.7 (2C), 135.1 (2C), 133.1 (2C), 126.8 (4C), 120.0 (4C), 118.9 (4C), 117.4 (4C), 56.6 (4C), 54.2 (4C), 40.2 (4C). HRMS (ESI): *m/z* calculated for C_42_H_50_GaN_12_O_12_S_2_^+^ (M^+^), 1047.2363; found, 1047.2369.

#### Radiolabeling

For ^67^Ga-labeling, a [^67^Ga]GaCl_3_ solution (2 μL, 0.1 MBq) was mixed with 0.1 M MES buffer (pH 5.5, 300 μL) and the precursor in DMSO (final 0.5 mM, 10 μL), and then the mixture was incubated at 90°C for 30 min. After cooling to room temperature, the mixture was purified by RP-HPLC on a Cosmosil C_18_ column (5C_18_-PAQ, 4.6 × 250 mm) with a solvent of H_2_O/MeCN/trifluoroacetic acid (TFA) [90:10:0.1 (0 min) to 60:40:0.1 (30 min)] as the mobile phase at a flow rate of 1.0 mL/min. The ^67^Ga-labeled compound was analyzed by analytical RP-HPLC.

For ^68^Ga-labeling, a [^68^Ga]GaCl_3_ solution (500 μL, 74 MBq) was mixed with 1.2 M acetate buffer (pH 4.0, 500 μL) and the precursor in DMSO (final 5 mM, 500 μL). The reaction mixture was then incubated at 90°C for 20 min. After cooling to room temperature, the mixture was purified by RP-HPLC on a Cosmosil C_18_ column (5C_18_-PAQ, 4.6 × 250 mm) with a solvent of H_2_O/MeCN/TFA [90:10:0.1 (0 min) to 60:40:0.1 (30 min)] as the mobile phase at a flow rate of 1.0 mL/min. The ^68^Ga-labeled compound was analyzed by analytical RP-HPLC.

### Measurement of partition coefficient

The experimental determination of partition coefficients was performed in 1-octanol and phosphate-buffered saline (PBS) (pH 7.4). 1-Octanol (3 mL) and PBS (3 mL) were pipetted into a 15-mL test tube containing [^67^Ga]Ga-US2 (50 kBq, 41 GBq/μmol). The test tube was vortexed for 2 min and centrifuged (4,000 ×*g*, 5 min). Aliquots (0.5 mL) from the 1-octanol and PBS phases were transferred into two test tubes for counting. The remaining PBS phase (1 mL), newly prepared 1-octanol (3 mL), and PBS (2 mL) were pipetted into a new test tube. The vortexing, centrifuging, and counting were repeated until consistent partition coefficient values were obtained (usually the sixth partition). The amount of radioactivity in each tube was measured with a γ counter (Wallac 1470 Wizard; PerkinElmer, Massachusetts, U.S.A.). The partition coefficient was calculated using the equation: log P_ow_ = log[count_1-octanol_/count_PBS_].

### Animals

All animal experiments were performed in accordance with our institutional guidelines and were approved by the Kyoto University Animal Care Committee. Male BALB/c-*nu*/*nu* nude mice and male ddY mice were purchased from Japan SLC (Shizuoka, Japan). Animals were administered materials without anesthesia. Animal sacrifice and blood collection were performed by decapitation. Animals were housed in a sterile environment with a 12-h light-dark cycle, fed standard chow, and had free access to water. All efforts were made to minimize suffering.

### Analysis of stability in mouse plasma

The blood (5 mL) was collected from ddY mice (male, 5 weeks old) and centrifuged (1,200 ×*g*, 10 min) in venous blood collection tubes (Becton, Dickinson and Company, New Jersey, U.S.A.). The plasma (200 μL) was separated and [^67^Ga]Ga-US2 (185 kBq, 149 GBq/μmol) was added to it. The solution was incubated at 37°C for 1 and 4 h. After the addition of MeCN (200 μL), it was centrifuged (10,000 ×*g*, 5 min). The supernatant was filtered with a Cosmonice Filter (S) (0.45 μm, 4 mm) (Nacalai Tesque), and the filtrate was analyzed by RP-HPLC. The analytical method of RP-HPLC was the same as written in the radiolabeling section.

### Cell culture

HT-29 and MDA-MB-231, which are human colorectal cancer cell lines and human breast cancer cell lines, respectively, were purchased from Sumitomo Dainippon Pharma (Osaka, Japan). RCC4 plus VHL (RCC4-VHL) and RCC4 plus vector alone (RCC4-VA), which are human renal cell carcinoma cell lines (RCC4) stably transfected with pcDNA3-VHL (VHL-expressing vector) and pcDNA3 (empty vector), respectively, were purchased from DS Pharma Biomedical (Osaka, Japan). Cells were maintained in Dulbecco’s modified Eagle’s medium (DMEM) (Nacalai Tesque) supplemented with 10% heat-inactivated fetal bovine serum (Thermo Fisher Scientific, Massachusetts, U.S.A.), 100 U/mL penicillin, and 100 μg/mL streptomycin at 37°C in an atmosphere containing 5% CO_2_.

### *In vitro* cell binding assay

HT-29, MDA-MB-231, RCC4-VHL, and RCC4-VA cells were incubated in 12-well plates (2 × 10^5^ cells/well) at 37°C in an atmosphere containing 5% CO_2_ for 24 h. After incubation, cells were then incubated at 37°C in an atmosphere containing 5% CO_2_ and 21% O_2_ (normoxic conditions) or 1% O_2_ (hypoxic conditions) for another 24 h. After removing the medium, [^67^Ga]Ga-US2 (18 kBq, 50−82 GBq/μmol) in the medium (1 mL) was added to each well inside the hypoxic chamber (miniMACS Anaerobic Workstation; Don Whitley Scientific, West Yorkshire, U.K.), and the plates were incubated under normoxic or hypoxic conditions for 2 h. Nonspecific binding was evaluated by the addition of acetazolamide (50 μM). After incubation, each well was washed with 1 mL of PBS (pH 7.4) (Nacalai Tesque), and the cells were lysed with 1 M NaOH (0.5 mL × 2). Radioactivity bound to cells was measured using a γ counter (PerkinElmer). The protein concentration was determined using BCA Protein Assay Kit (Thermo Fisher Scientific).

### Tumor model

Under anesthesia with isoflurane (2% in an air mixture), BALB/c-*nu*/*nu* nude mice (male, 5 weeks old) were subcutaneously inoculated with MDA-MB-231 cells (1 × 10^7^ cells/mouse), in 150 μL of DMEM and Geltrex (Thermo Fisher Scientific) at a 1:1 ratio, in the left flank. Fifteen days later, HT-29 cells (1 × 10^7^ cells/mouse) were also subcutaneously injected into the right flank of MDA-MB-231 tumor-bearing mice. For the PET/CT and pharmacological therapy studies, BALB/c-*nu*/*nu* nude mice were subcutaneously inoculated with only HT-29 cells (5 × 10^6^ cells/mouse) in the right flank. All efforts were made to minimize suffering.

### Biodistribution study in model mice

A saline solution (100 μL) of [^67^Ga]Ga-US2 (28 kBq, 85 GBq/μmol) was directly injected into the tail vein of HT-29 and MDA-MB-231 tumor-bearing mice (n = 5 each time). For *in vivo* competition studies, tumor-bearing mice were injected with [^67^Ga]Ga-US2 (28 kBq, 85 GBq/μmol) and acetazolamide (10 mg/kg) in saline (100 μL) concurrently. The mice were sacrificed at 10, 30, 60, and 120 min postinjection. The blood, spleen, pancreas, stomach, intestines, kidneys, liver, heart, lungs, brain, HT-29 tumor, MDA-MB-231 tumor, and muscle were collected. Each organ was weighed and the radioactivity was measured using a γ counter (PerkinElmer). The % injected dose/g of samples was calculated by comparing the sample counts with the count of the initial dose.

### PET/CT

A solution of [^68^Ga]Ga-US2 (1.1−3.5 MBq, 46−216 GBq/μmol) in saline (100 μL) was directly injected into the tail vein of HT-29 tumor-bearing mice. PET and CT images were collected using the G8 PET/CT system (PerkinElmer) (PET conditions: 10 min × 1 frame; CT conditions: accurate full angle mode in 60 kV/615 μA) at 60 and 120 min postinjection. Cross-calibration between the dose calibrator and the imaging systems was performed according to the manufacturer’s specifications. The images were acquired as static whole-body scans using G8 acquisition software and reconstructed with maximum-likelihood expectation maximization (MLEM) according to a previous report [53]. Acquired PET and CT data were analyzed using PMOD software (Version 3.3; PMOD Technologies, Zürich, Switzerland).

### Pharmacological therapy

[^nat^In]In-US2 was solubilized in 37.5% PEG400/12.5% EtOH/50% saline prior to administration. A dose of 50 mg/kg [^nat^In]In-US2 or vehicle was administered via intraperitoneal injection using a volume of 100 μL/mouse three times a week for 2 weeks (n = 12). The tumor volume and body weight were measured three times a week for 4 weeks after the injection of [^nat^In]In-US2. The tumor volume was calculated using the formula: V = [length × (width)^2^]/2. The initial tumor volumes (on day 0) for the [^nat^In]In-US2 and vehicle groups were 72.6 ± 7.2 and 74.4 ± 8.0 mm^3^ (mean ± standard error), respectively. Mice were euthanized when the tumor volume reached 1,000 mm^3^ or the body weight had decreased by over 20% from the original weight.

### Statistical analysis

All data were analyzed with GraphPad Prism or Microsoft Excel. Differences at the 95% confidence level (P < 0.05) were considered significant.

## Results

### Chemistry and radiolabeling

[^nat^Ga]Ga-US2 was prepared by reacting the precursor with [^nat^Ga]Ga(NO_3_)_3_·nH_2_O in MES buffer (0.1 M, pH 5.5) at a 38% yield. The yield suggested that [^nat^Ga]Ga-US2 was dissolved in the solvent to some extent. The ^1^H NMR, ^13^C NMR, and HRMS were consistent with the assigned structures.

**Fig 2:**
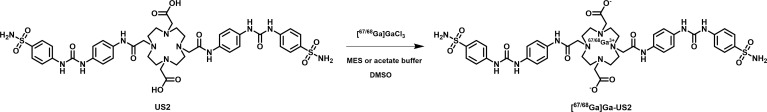
Radiolabeling of [^67/68^Ga]Ga-US2.

^67^Ga-labeling was carried out by incubating the precursor with [^67^Ga]GaCl_3_ in 0.1 M MES buffer (pH 5.5) at 90°C (Fig 2). After purification by RP-HPLC, [^67^Ga]Ga-US2 was obtained with high radiochemical purity (> 95%) as determined by RP-HPLC. [^67^Ga]Ga-US2 was synthesized at a 51% radiochemical yield (decay-corrected). The ^68^Ga-labeling reaction was performed by incubating the precursor with [^68^Ga]GaCl_3_ in 1.2 M acetate buffer (pH 4.0) at 90°C (Fig 2). [^68^Ga]Ga-US2 was synthesized at a 30% radiochemical yield (decay-corrected) with over 95% radiochemical purity. The radiochemical identity of the ^67/68^Ga complex was verified by comparative RP-HPLC using the corresponding ^nat^Ga complex as a reference. The retention times between the ^67/68^Ga-labeled compound (22.8/22.7 min) and corresponding ^nat^Ga complex (22.7 min) suggest that the desired ^67/68^Ga-labeled ureidosulfonamide derivatives were successfully synthesized.

### Hydrophilicity and *in vitro* stability

**Fig 3:**
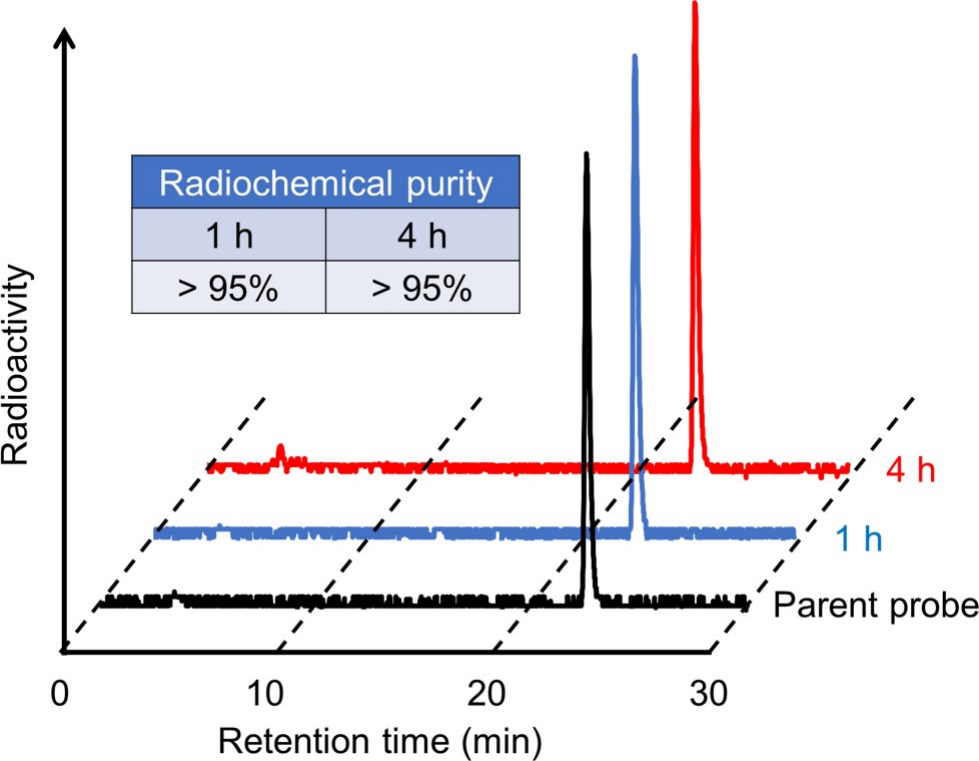
Stability of [^67^Ga]Ga-US2 in mouse plasma. Radiochemical purity of [^67^Ga]Ga-US2 after 1- and 4 h-incubation in mouse plasma.

We examined the hydrophilicity of radiogallium-labeled US2, and the log P_ow_ value for [^67^Ga]Ga-US2 was −1.79 ± 0.02. In addition, the *in vitro* stability of [^67^Ga]Ga-US2 was evaluated by incubating it in the mouse plasma at 37°C for pre-determined times. Almost all [^67^Ga]Ga-US2 existed as an intact form (> 95%) until 4 h *in vitro* (Fig 3).

### *In vitro* cell binding

**Fig 4:**
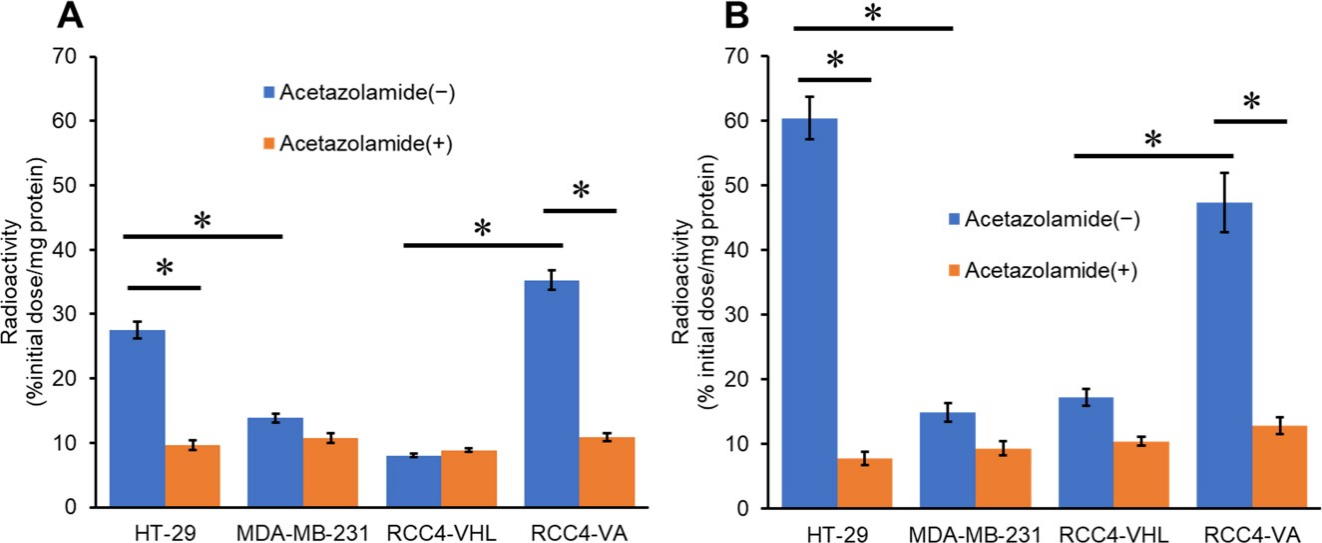
Cell binding assay with [^67^Ga]Ga-US2 under normoxic (A) and hypoxic (B) conditions. Values are expressed as the mean ± standard deviation of three independent experiments. *P < 0.01 (two-tailed Student’s *t*-test).

A cell binding assay was performed to evaluate the *in vitro* CA-IX affinity of [^67^Ga]Ga-US2 under both normoxic and hypoxic conditions (Fig 4). According to our previous study, HT-29 and RCC4-VA cell lines were used as CA-IX high-expressing cells, while MDA-MB-231 and RCC4-VHL cell lines were used as CA-IX low-expressing ones, under both normoxic and hypoxic conditions [46]. The binding of [^67^Ga]Ga-US2 under normoxic conditions to CA-IX high-expressing (HT-29 and RCC4-VA) cells (27.5 and 35.3% initial dose/mg protein, respectively) was significantly greater than that to CA-IX low-expressing (MDA-MB-231 and RCC4-VHL) cells (13.9 and 8.04% initial dose/mg protein, respectively), indicating the selectivity of [^67^Ga]Ga-US2 for CA-IX. These values partially corresponded with relative concentrations of CA-IX in the cell lines determined by Western blotting analyses (CA-IX/GAPDH = 1.75, 0.20, 0.18, and 0.54 for HT-29, MDA-MB-231, RCC4-VHL, and RCC4-VA, respectively) [46]. In addition, the addition of acetazolamide, a classical CA inhibitor, significantly blocked binding to the CA-IX high-expressing cells (9.67 and 10.8% initial dose/mg protein), suggesting the *in vitro* CA-specificity (Fig 4A). In response to hypoxia, the binding of [^67^Ga]Ga-US2 to HT-29 cells was markedly enhanced (27.5 to 60.4% initial dose/mg protein), which was consistent with CA-IX expression (CA-IX/GAPDH = 1.75 to 3.32) [46], suggesting that [^67^Ga]Ga-US2 could detect hypoxic regions of tumors. The selective binding to CA-IX high-expressing cells was also observed under hypoxic conditions, and its binding was blocked by acetazolamide (Fig 4B).

### *In vivo* tumor uptake

**Fig 5:**
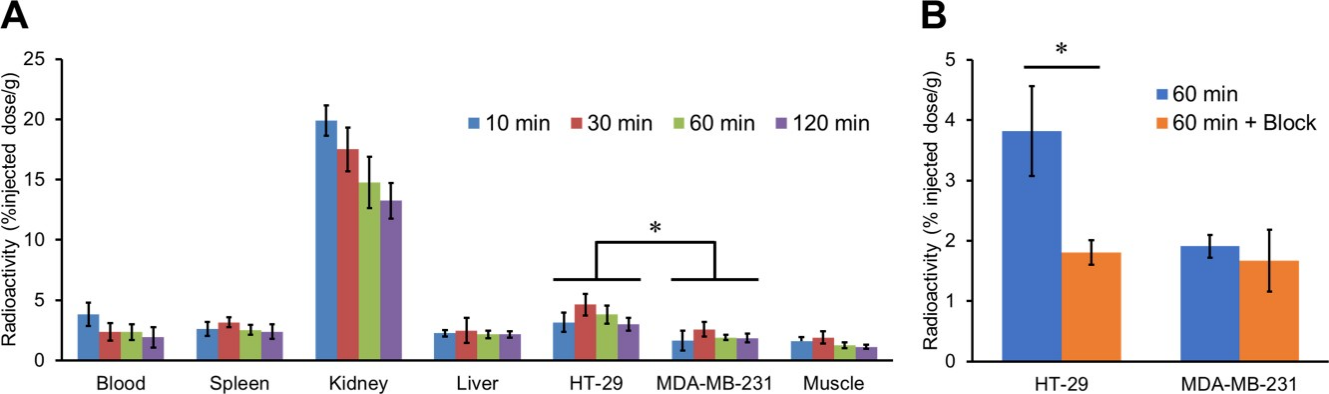
*In vivo* biodistribution study with [^67^Ga]Ga-US2 in HT-29 and MDA-MB-231 tumor-bearing mice. (A) Radioactivity of representative extracted organs and tissues after the intravenous injection of [^67^Ga]Ga-US2 in the HT-29 and MDA-MB-231 tumor-bearing mice (n = 5). (B) Radioactivity of the extracted tumor after the intravenous injection of [^67^Ga]Ga-US2 with or without acetazolamide (10 mg/kg mouse) in the HT-29 and MDA-MB-231 tumor-bearing mice. Values are expressed as the mean ± standard deviation of five mice. *P < 0.05 each time (two-tailed Student’s *t*-test).

A biodistribution study was carried out to assess the *in vivo* uptake into the CA-IX-expressing tumor in the cancer model mice (Fig 5 and S1 Table). According to our previous report, HT-29 and MDA-MB-231 tumors were used as CA-IX high- and low-expressing ones, respectively [46]. [^67^Ga]Ga-US2 showed significantly higher accumulation in the HT-29 tumor (2.98−4.63% injected dose/g) than that in the MDA-MB-231 tumor (1.64−2.58% injected dose/g) at all evaluated timepoints, indicating the selective accumulation in CA-IX high-expressing tumors. Among normal organs, marked kidney uptake was observed (13.3−19.9% injected dose/g). Radioactivity in the HT-29 tumor at 60 min postinjection (3.81% injected dose/g) was significantly decreased by the coinjection of acetazolamide (1.80% injected dose/g), indicating the *in vivo* specificity of [^67^Ga]Ga-US2 for CA (Fig 5B). Moreover, both the HT-29 tumor/blood and HT-29 tumor/muscle ratios remained above 1.5 after 30 min postinjection, suggesting favorable properties for *in vivo* imaging of solid tumors (S1 Table).

### PET/CT

**Fig 6:**
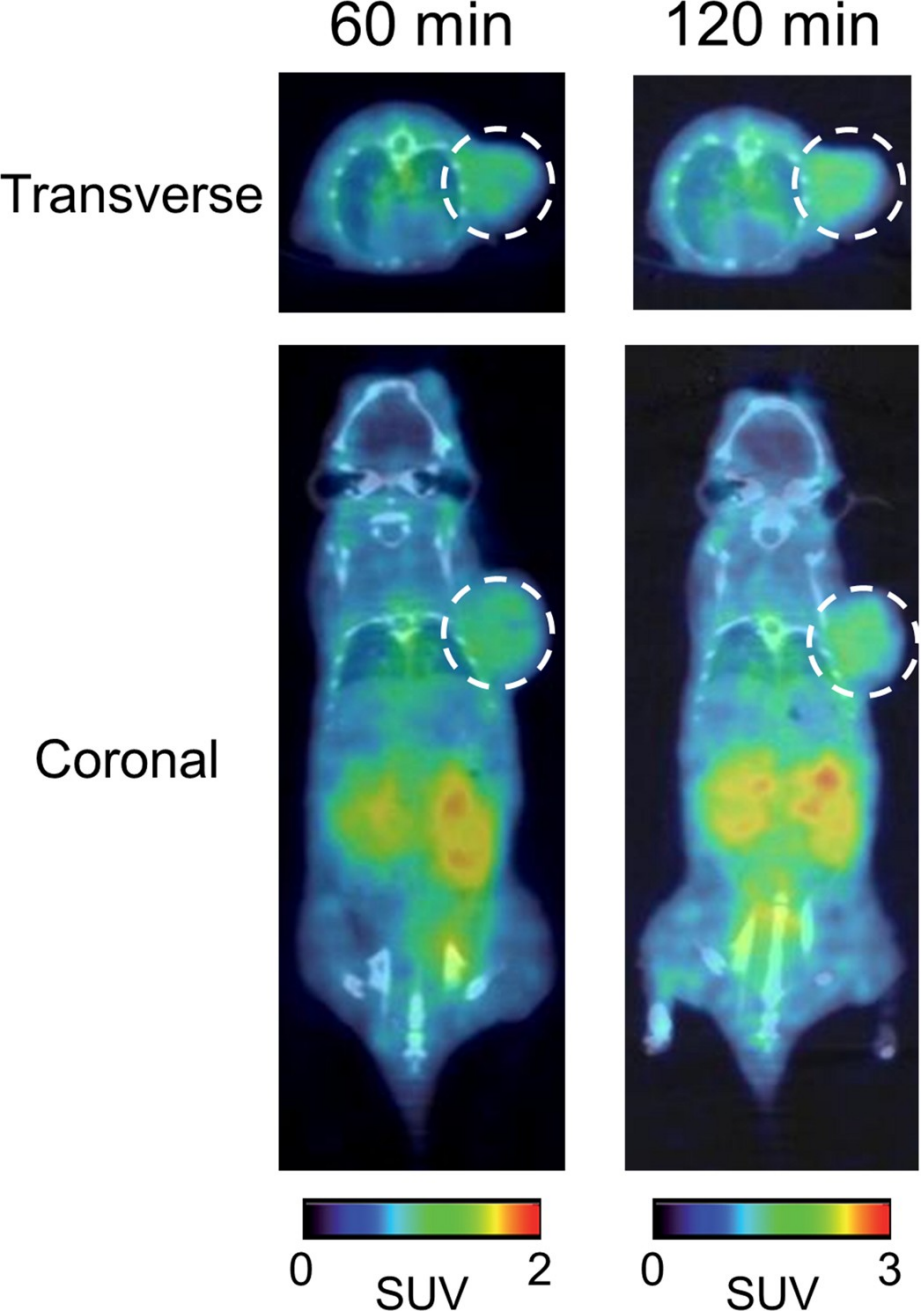
PET/CT of an HT-29 tumor-bearing mouse. PET/CT images of an HT-29 tumor-bearing mouse with [^68^Ga]Ga-US2 at 60 and 120 min postinjection. White dashed circles indicate the tumor.

PET/CT study with [^68^Ga]Ga-US2 targeting CA-IX high-expressing tumors was performed (Fig 6). [^68^Ga]Ga-US2 clearly visualized the HT-29 tumors at 60 and 120 min postinjection probably due to its favorable tumor/blood and tumor/muscle ratios demonstrated in the biodistribution study (S1 Table), while no marked difference in radioactivity biodistribution between 60 and 120 min postinjection was observed. However, a high level of radioactivity accumulation in the kidneys and bladder was observed, suggesting renal excretion of [^68^Ga]Ga-US2. Radioactivity biodistribution on PET corresponded with the results of the biodistribution study with [^67^Ga]Ga-US2 (Fig 5).

### Pharmacological therapy

**Fig 7:**
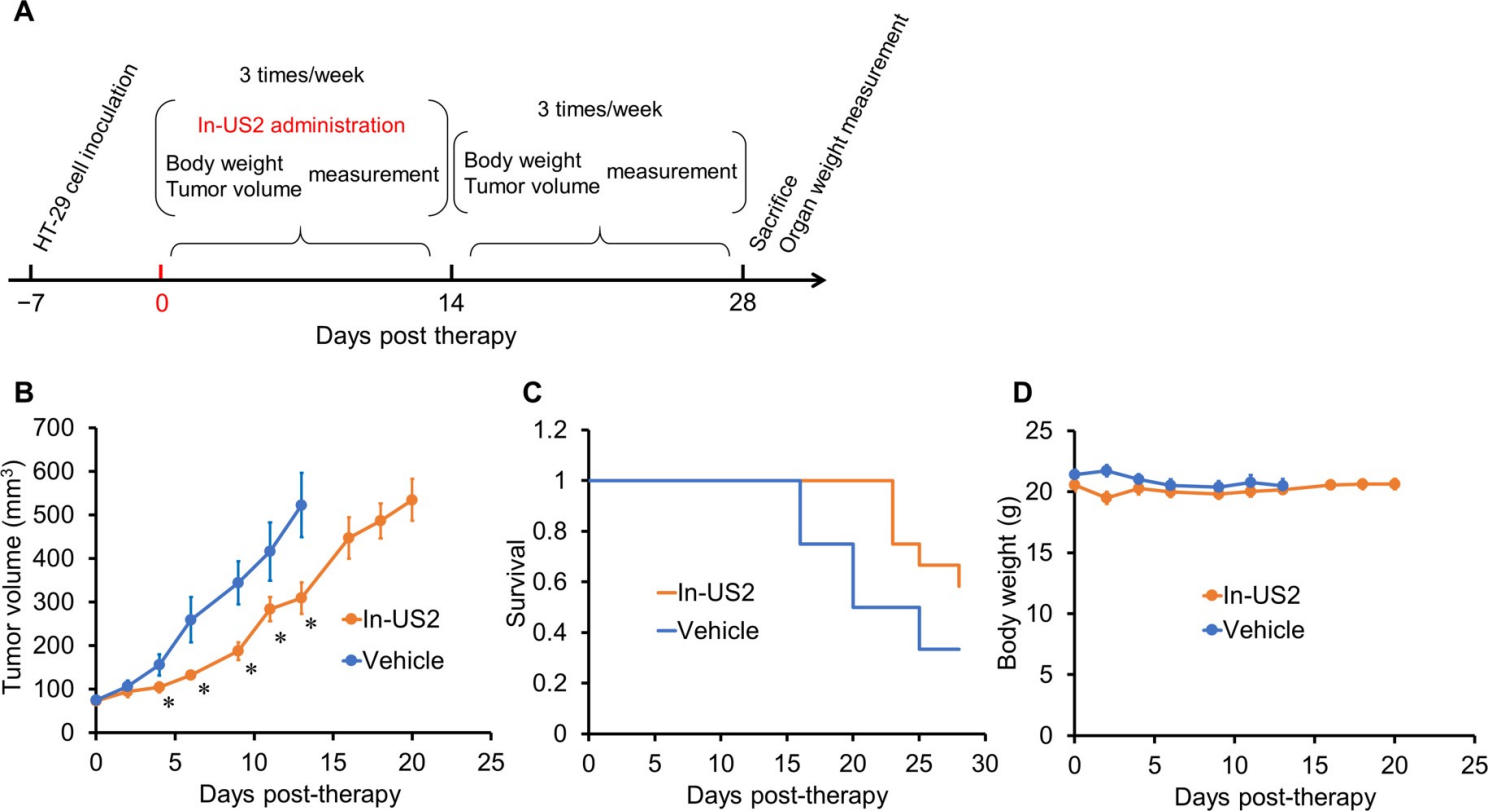
Pharmacological therapy with [^nat^In]In-US2 for HT-29 tumor-bearing mice. (A) Schematic diagram of treatments and measurements. (B) Tumor growth inhibition by [^nat^In]In-US2. Values are expressed as the mean ± standard error of twelve mice. (C) The effect of treatment with [^nat^In]In-US2 using twelve mice based on Kaplan-Meier survival curve analysis. (D) Change in body weight of tumor-bearing mice. Values are expressed as the mean ± standard error of twelve mice. *P < 0.05 compared with the vehicle group (two-tailed Student’s *t*-test).

Pharmacological therapy with [^nat^In]In-US2 targeting CA-IX high-expressing tumors was carried out (Fig 7). The injected dose of [^nat^In]In-US2 was 50 mg/kg mouse and administered via intraperitoneal injection. The therapeutic effect was evaluated by monitoring the tumor volume in mice using a caliper three times a week. As shown in Fig 7B, the tumor volume values for mice administered [^nat^In]In-US2 were markedly lower than those with vehicle, indicating that pharmacological therapy with 50 mg/kg [^nat^In]In-US2 delayed tumor growth. Mouse survival was prolonged by [^nat^In]In-US2, suggesting the therapeutic effect of [^nat^In]In-US2 (Fig 7C). The toxicity of [^nat^In]In-US2 was evaluated by monitoring the body weight. No marked change in the body weight of mice was observed, indicating the low toxicity of [^nat^In]In-US2 (Fig 7D). Moreover, on day 28, mice were sacrificed, and their spleen, kidneys, and liver were removed and weighed (S1 Fig). No marked difference in organ weight between mice treated with [^nat^In]In-US2 and vehicle was observed, suggesting no critical toxic effect of the ^nat^In complex on these organs.

## Discussion

We previously reported the utility of bivalent ureidosulfonamide (US2) for CA-IX-targeting radiotheranostics. In the present study, we applied this US2 platform to PET imaging and pharmacological therapy targeting CA-IX high-expressing tumors.

Labeling with ^67/68^Ga was performed in MES or acetate buffer at a favorable radiochemical yield. A previous report suggested that a 1,4,7,10-tetraazacyclododecane-1,4,7,10-tetraacetic acid (DOTA)-linked agent, DOTATOC, showed a varying biodistribution depending on the conjugated radiometal (^67^Ga, ^90^Y, and ^111^In) [54]. Moreover, several geometries of In-DOTA and Ga-DOTA were reported [55], suggesting that [^67/68^Ga]Ga-US2 could adopt a different geometry from [^111^In]In-US2. Therefore, we evaluated the fundamental property of [^67/68^Ga]Ga-US2 for the CA-IX imaging agent to compare it with the corresponding ^111^In complex. First, the log P_ow_ value for [^67^Ga]Ga-US2 was −1.79 ± 0.02, while that for [^111^In]In-US2 was −2.81 ± 0.01, indicating the lower hydrophilicity of [^67/68^Ga]Ga-US2 than [^111^In]In-US2. [^67^Ga]Ga-US2 showed high stability in mouse plasma like [^111^In]In-US2 (Fig 3), indicating that replacement of the radiometal in US2 did not affect the stability of the complex. In addition, [^67^Ga]Ga-US2 showed the ability to target CA-IX high-expressing cells *in vitro* (Fig 4) and tumors *in vivo* (Fig 5). However, in the biodistribution assay, HT-29 tumor uptake of [^67^Ga]Ga-US2 at 60 min postinjection (3.81% injected dose/g) was slightly lower than that of [^111^In]In-US2 (4.57% injected dose/g), suggesting the influence of the metal change on their biodistribution. A lower uptake than [^111^In]In-US2 at 60 min postinjection was also observed in the kidney and liver ([^67^Ga]Ga-US2, 14.8 and 2.17% injected dose/g; [^111^In]In-US2, 18.6 and 4.17% injected dose/g, respectively), and faster clearance from the blood and muscle was shown ([^67^Ga]Ga-US2, 2.36 and 1.25% injected dose/g; [^111^In]In-US2, 4.17 and 1.62% injected dose/g, respectively, at 60 min postinjection). Many groups have reported CA-IX imaging probes for PET/SPECT, and most of them, in addition to us, used HT-29 tumor-bearing mice as the CA-IX high-expressing tumor models. [^111^In/^67^Ga]In/Ga-US2 showed greater HT-29 tumor uptake than probes reported by other groups, including ^68^Ga-labeled benzensulfonamides [26, 27, 31, 46–52]. Recently, SKRC-52 was used as a CA-IX high-expressing cell line with a greater CA-IX expression than HT-29 [56]. Although ^111^In, ^64^Cu, and ^99m^Tc-labeled probes exhibited higher accumulation in SKRC-52 tumors as compared with HT-29 tumor accumulation of [^111^In/^67^Ga]In/Ga-US2 [24, 29, 33], these accumulations are not comparable. [^67^Ga]Ga-US2 showed fast clearance from the blood and muscle, suggesting favorable pharmacokinetics for *in vivo* imaging of solid tumors. The rapid pharmacokinetics of radiogallium-labeled US2 due to its low molecular weight are significant for ^68^Ga-PET, while imaging with antibody-based probes showing slow clearance from the blood pool requires longer-half-life radionuclides, such as ^124^I, ^131^I, ^111^In, and ^89^Zr [18–22]. However, [^67^Ga]Ga-US2 markedly accumulated in the kidney and lung as well as [^111^In]In-US2, which might lead to unclear imaging of such types of cancer. Their high accumulation might be due to the expression of other CA isozymes on the cell surface in these organs [57], since radioactivity accumulation in them was decreased by acetazolamide (S1 Table). Blocking of the transmembrane isozymes in normal organs by nonradioactive CA ligands at appropriate doses may improve the contrast of the tumor image, since the number of CA molecules in normal organs might be lower than in tumors [24]. PET imaging of mice provided a clear image of the HT-29 tumor; however, a high level of radioactivity in the kidneys was observed, as demonstrated in the biodistribution study (Fig 6). Moreover, SPECT/CT studies with [^67^Ga]Ga-US2 using HT-29 and MDA-MB-231 tumor-bearing mice were also carried out to evaluate CA-IX selectivity of radiogallium-labeled US2 (S2 Fig). The SPECT image at 60 min postinjection showed greater radioactivity accumulation in the HT-29 tumor than the MDA-MB-231 tumor, indicating CA-IX-selective tumor visualization of radiogallium-labeled US2. SPECT with [^67^Ga]Ga-US2 showed a similar pharmacokinetics profile to that of PET with [^68^Ga]Ga-US2. SPECT with [^67^Ga]Ga-US2 suggests the efficacy of the other modality using US2, reinforcing the utility of the US2 platform. These results with [^67/68^Ga]Ga-US2 indicate that [^68^Ga]Ga-US2 may be a useful CA-IX-targeting PET probe and that the US2 platform can provide a CA-IX-PET imaging strategy by conjugating with ^68^Ga.

Recently, ^68^Ga has been attracting much attention as a useful radionuclide for PET imaging because it can be easily obtained from a commercial generator. However, its short half-life (68 min) limits the selection of targeting ligands. High-molecular-weight compounds such as antibodies, generally showing long retention in the blood pool, are inappropriate for ^68^Ga-PET, since it is difficult to reach a high signal-to-background ratio at an early time post-administration. Radiogallium-labeled US2 showed rapid pharmacokinetics; however, its tumor accumulation was less than clinically utilized tumor-imaging probes, such as [^68^Ga]Ga-PSMA-11 targeting prostate-specific membrane antigen [58]. We recently identified a small-molecule CA-IX ligand, imidazothiadiazole sulfonamide (IS), with favorable properties for CA-IX imaging [59]; thus, a small-molecule ^68^Ga-PET probe based on IS may provide a clearer image of CA-IX-expressing tumors than [^68^Ga]Ga-US2.

For the therapeutic application of US2, we synthesized [^nat^In]In-US2 and administered it to HT-29 tumor-bearing mice. Nonradioactive indium was conjugated with US2, because [^111^In]In-US2 showed greater tumor accumulation than [^67^Ga]Ga-US2 in the biodistribution study. *In vivo* CA-IX inhibition by sulfamate, sulfonamide, and coumarin inhibitors has been shown to reverse the effect of tumor acidification, leading to a delay in tumor growth, while the effects depended on the treatment schedule, administered dose, and tumor cell line [23, 30, 38, 40, 60, 61]. The unsubstituted sulfonamides and their bioisosteres, including ureidosulfonamide, bind to the Zn^2+^ ion of the enzyme by substituting the non-protein zinc ligand, such as H_2_O, to generate a tetrahedral adduct, leading to the inhibition of enzyme activity [5]. [^nat^In]In-US2 therapy showed tumor growth delay and survival prolongation in HT-29 tumor-bearing mice, indicating therapeutic effects on the HT-29 tumor (Fig 7C and 7D). Meanwhile, no marked change in the whole body or organ weight by [^nat^In]In-US2 was observed, suggesting no critical toxicity of [^nat^In]In-US2 (Figs 7E and S1). Therefore, the US2 platform can also provide a tumor therapeutic application targeting CA-IX.

Pharmacological therapy with [^nat^In]In-US2 targeting CA-IX showed limited therapeutic effects: no remission or shrinkage of tumors. Combination therapy with anti-tumor drugs, such as cisplatin, doxorubicin, and fluorouracil has shown greater therapeutic effects on tumors [62–64]. It is generally accepted that hypoxia in tumors often causes resistance to anti-tumor drugs through the HIF-1 cascade; therefore, the inhibition of CA-IX, closely associated with hypoxia, can enhance the effect of anti-tumor drugs, leading to a decrease in the dose of anti-tumor drugs for therapy, which may help realize safer cancer treatments. Thus, [^nat^In]In-US2 administration may enhance the effects of conventional therapies.

## Conclusions

According to our previous study using ^111^In and ^90^Y complexes based on a bivalent ureidosulfonamide scaffold ([^111^In/^90^Y]In/Y-US2) for cancer radiotheranostics targeting CA-IX, we applied the US2 platform to ^68^Ga-PET imaging ([^68^Ga]Ga-US2) of and pharmacological therapy ([^nat^In]In-US2) for CA-IX high-expressing tumors. Fundamental studies with [^67^Ga]Ga-US2 revealed specific binding of radiogallium-labeled US2 to CA-IX high-expressing cells *in vitro* and tumors *in vivo*; moreover, PET imaging with [^68^Ga]Ga-US2 clearly visualized CA-IX high-expressing tumors in mice. In addition, pharmacological therapy with [^nat^In]In-US2 using CA-IX high-expressing tumor-bearing mice delayed tumor growth and prolonged survival. The results suggest that US2, which can conjugate with various metals, may be useful as a theranostic platform utilizing CA-IX.

## Supporting information

S1 File Supporting materials and methods. (DOCX)

S1 Table Radioactivity of extracted organs after intravenous injection of [^67^Ga]Ga-US2 in HT-29 and MDA-MB-231 tumor-bearing mice. Values are expressed as the % injected dose per gram of tissue. Each value is the mean ± standard deviation of five animals at each interval. *Coinjection of acetazolamide (10 mg/kg). ^†^Values are expressed as the % injected dose.
(XLSX)

S1 Fig Spleen, kidney, and liver weight of mice treated with [^nat^In]In-US2 or vehicle at the end of the therapeutic experiment. (TIF)

S2 Fig SPECT/CT images of an HT-29 and MDA-MB-231 tumor-bearing mouse with [^67^Ga]Ga-US2 at 60 min postinjection. (TIF)
