# Supplementary material for: PET imaging and pharmacological therapy targeting carbonic anhydrase-IX high-expressing tumors using US2 platform based on bivalent ureidosulfonamide
Source: PLoS One. 2020 Dec 9;15(12):e0243327. doi: 10.1371/journal.pone.0243327 (PMC7725290; doi:10.1371/journal.pone.0243327)
Supplement: S1 File — (DOCX) [file pone.0243327.s001.docx]

**S1 File. Supporting Materials and Methods**

**Single photon emission computed tomography (SPECT)/CT**

A saline solution (150 μL) of [^67^Ga]Ga-US2 (8.9 MBq, 88 GBq/μmol) was directly injected into the tail vein of HT-29 and MDA-MB-231 tumor-bearing mice. The mice were anesthetized by isoflurane (2% in an air mixture). SPECT and CT images were collected using the U-SPECT-II system (MILabs, Utrecht, the Netherlands) with 1.0-mm pinhole collimators (SPECT conditions: 60 min × 1 frame; CT conditions: accurate full angle mode in 60 kV/615 μA) at 60 min postinjection. SPECT images were reconstructed using the ordered subset expectation maximization method. Acquired SPECT and CT data were analyzed using PMOD software (Version 3.3; PMOD Technologies, Zürich, Switzerland).
