# Supplementary figures and images for: PET imaging and pharmacological therapy targeting carbonic anhydrase-IX high-expressing tumors using US2 platform based on bivalent ureidosulfonamide
Source: PLoS One. 2020 Dec 9;15(12):e0243327. doi: 10.1371/journal.pone.0243327 (PMC7725290; doi:10.1371/journal.pone.0243327)

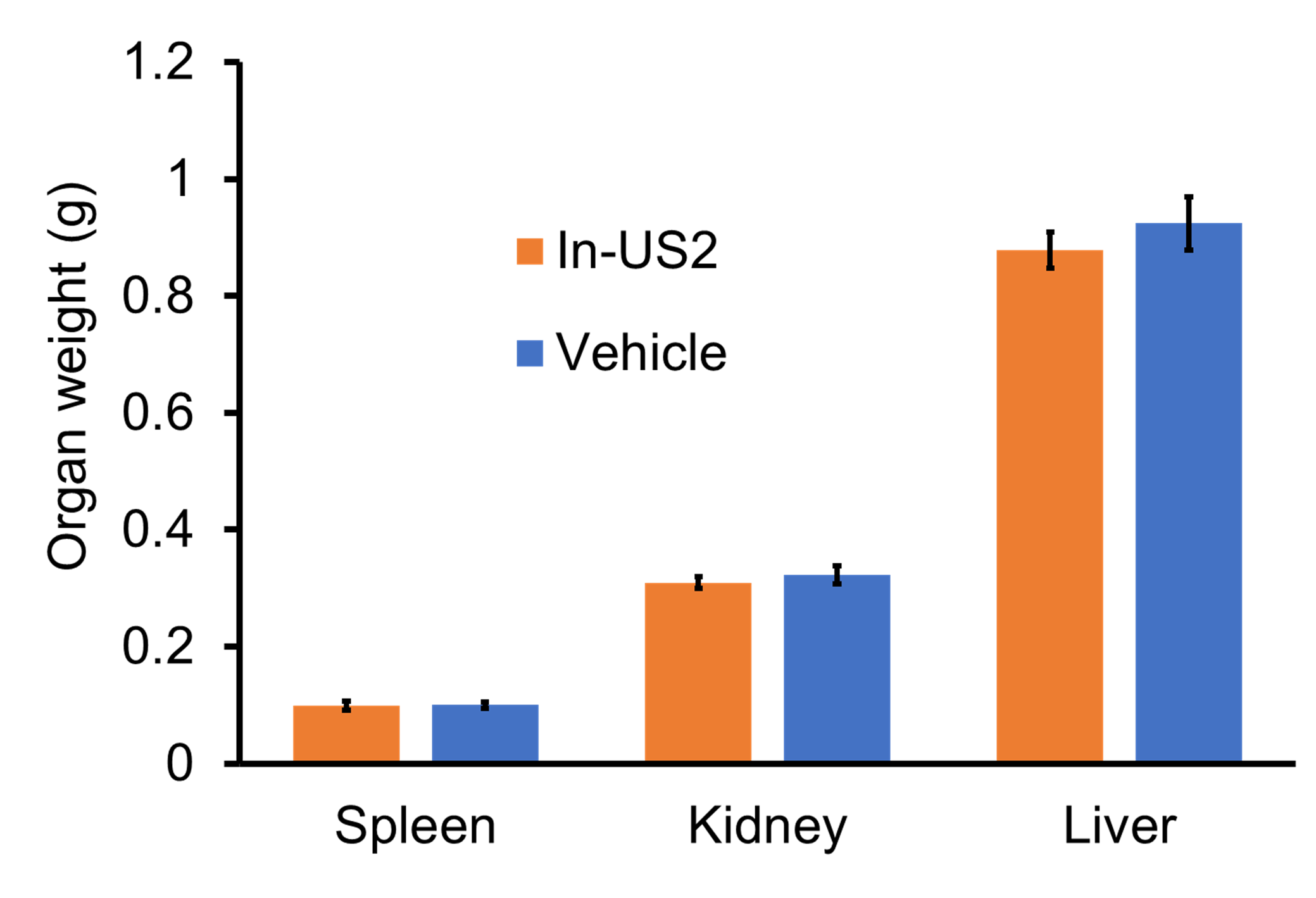

Supplement: S1 Fig — (TIF) [file pone.0243327.s003.tif]

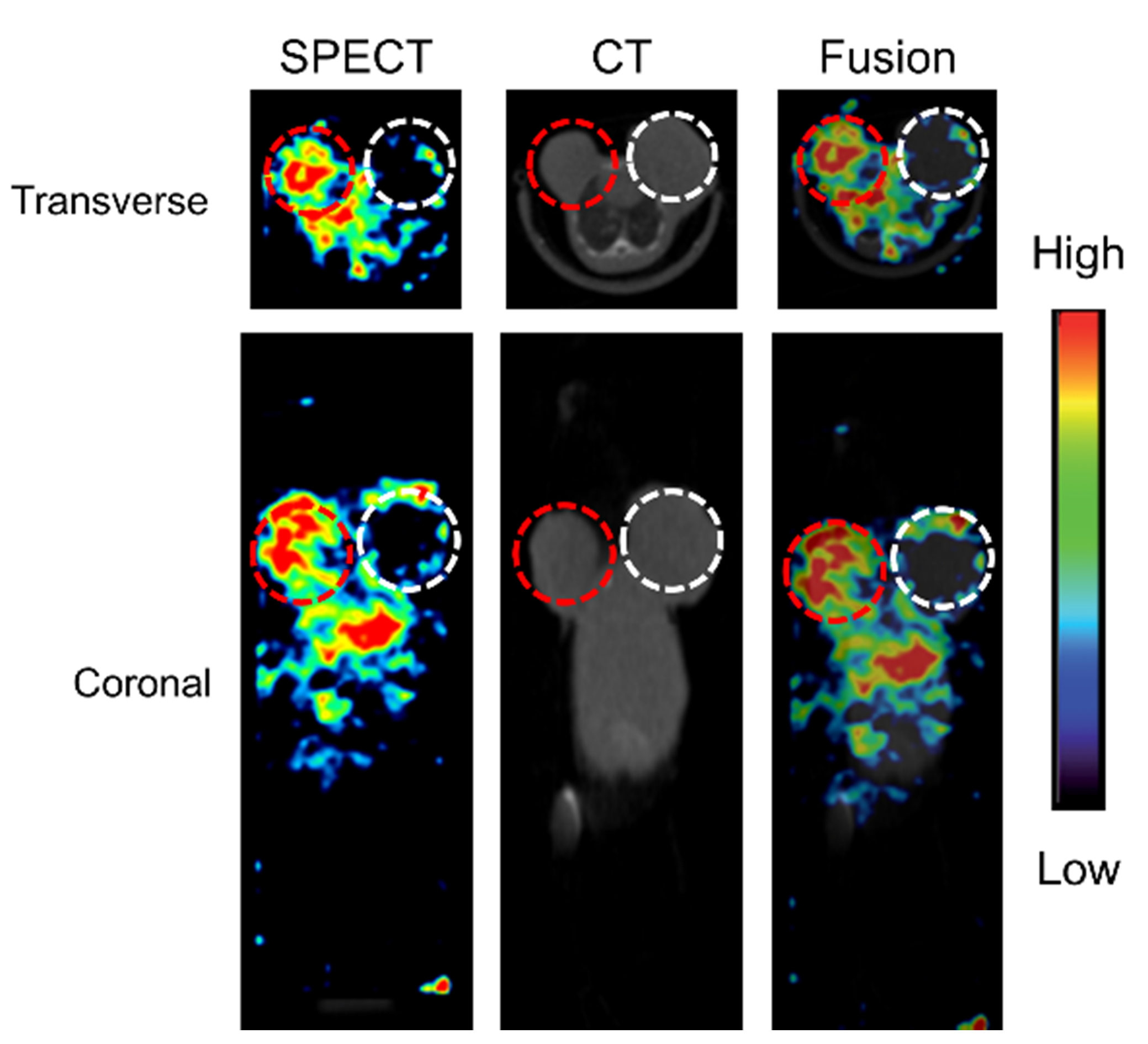

Supplement: S2 Fig — (TIF) [file pone.0243327.s004.tif]
